# Behavioral phenotypes of impulsivity related to the *ANKK1 *gene are independent of an acute stressor

**DOI:** 10.1186/1744-9081-4-54

**Published:** 2008-11-24

**Authors:** Melanie J White, C Phillip Morris, Bruce R Lawford, Ross McD Young

**Affiliations:** 1 Institute of Health and Biomedical Innovation, Queensland University of Technology, Kelvin Grove, Brisbane, Queensland 4059, Australia; 2 Department of Psychiatry, Royal Brisbane and Women's Hospital, Butterfield Street, Herston, Brisbane, Queensland 4029, Australia

## Abstract

**Background:**

The A1 allele of the *ANKK1 Taq*IA polymorphism (previously reported as located in the D2 dopamine receptor (DRD2) gene) is associated with reduced DRD2 density in the striatum and with clinical disorders, particularly addiction. It was hypothesized that impulsivity represents an endophenotype underlying these associations with the *Taq*IA and that environmental stress would moderate the strength of the gene-behavior relationship.

**Methods:**

*Taq*IA genotyping was conducted on 72 healthy young adults who were randomly allocated to either an acute psychosocial stress or relaxation induction condition. Behavioral phenotypes of impulsivity were measured using a card-sorting index of reinforcement sensitivity and computerized response inhibition and delay discounting tasks.

**Results:**

Separate analyses of variance revealed associations between the A1 allele and two laboratory measures of impulsivity. The presence of the *Taq*IA allele (A1+) was associated with slower card-sorting in the presence of small financial reinforcers, but was overcome in a second administration after either a five-minute rest or psychosocial stress induction. A1+ participants also demonstrated significantly poorer response inhibition and faster response times on a computerized stop inhibition task, independent of acute stress exposure.

**Conclusion:**

These findings indicate the A1 allele is associated with an endophenotype comprising both a "rash impulsive" behavioral style and reinforcement-related learning deficits. These effects are independent of stress.

## Background

The phenotypes of multiple psychiatric disorders involve features of impulsivity including attention deficit hyperactivity disorder (ADHD), bipolar disorder, mania, bulimia nervosa, several personality disorders, schizophrenia and substance dependence [1]. Impulsivity is also implicated in several forms of aggression and violence, sexual impulsivity, binge eating, obesity, self-harm and suicidal behavior [2]. As such, the study of factors that may contribute to impulsivity is fundamental to understanding and treating maladaptive human behavior.

Dopamine is integral to leading theories of an impulsive personality phenotype [3,4] and plays a crucial role in brain reinforcement circuits [2,5]. Personality theories of impulsivity also consistently emphasize the role of genetics, reflecting evidence from twin studies of high heritability on self-report measures, including sensation seeking (55% [6]), novelty seeking (40% [7]) and "rash/unplanned" impulsivity (15–40% [8]). Subsequently, genes associated with brain dopaminergic activity have been commonly studied candidates. The presence of the A1 allele of the *Taq*IA polymorphism (rs1800497) in the *ANKK1 *gene [9] (i.e., A1A1 and A1A2 genotypes) has been associated with reduced D2 dopamine receptor (DRD2) density in key structures linked to brain reinforcement, particularly in the striatum. This association has been confirmed by both *in vitro *[10,11] and *in vivo *positron emission tomography (PET) studies [12,13]. The *Taq*IA had been historically described as residing in the D2 dopamine receptor (DRD2) gene but has more recently been referred to as being within the *ANKK1 *[9]. In addition to these biological associations, a strong body of evidence suggests the involvement of the A1 allele in a range of behavioral disorders characterized by impulsivity, including severe alcohol and other substance abuse, obesity, pathological gambling and ADHD [14-16]. While A1 allele prevalence has been found to differ between ethnic populations [17], raising the issue of population stratification effects, research has shown that even across diverse ethnic groups there are still robust associations of the *Taq*IA with behavior [18]. Associations with addictive disorders have led to the hypothesis that *ANKK1 *is a reinforcement gene and those with the A1 allele are more likely to manifest brain reinforcement mechanism deficits than those without this allele [2,5]. Specifically, it is suggested that in an effort to compensate for inherited dopaminergic system deficiencies, individuals may seek to stimulate the mesocorticolimbic circuits and experience heightened reinforcement related to behaviors that increase brain dopamine levels (such as substance use), contributing to impulsive behavior. More direct evidence in rats supports an association between reduced D2 receptor availability in the ventral striatum and trait reinforcer-oriented impulsivity [19].

In behavioral genetics, simple Mendelian genetic influences are rare, with most traits reflecting the interplay of genes and environment [20]. In particular, the *ANKK1 *conferred risk appears to be conveyed via an interaction of A1 allelic risk and environmental stress [21-25]. For example, in a sample of alcoholic patients, negative life events and a harm avoidant personality were associated with severity of alcohol dependence in those carrying the A1 allele (A1+) but not in A1- patients [23]. Acute environmental stress is associated with impulsive behavior [26-28] and can increase dopamine neurotransmission in humans [29]. Further, recent PET scan findings suggest acute stress-induced striatal dopamine release may be greater in individuals at risk of psychosis. A significant decrease in [^11^C]raclopride binding potential (indicative of dopamine release), particularly in the ventral striatum, was reported for seven healthy students identified as at risk for psychosis due to elevated scores for physical anhedonia/negative schizotypy. In contrast, no such effect was found for 10 controls and 9 healthy adults scoring highly on perceptual aberrations/positive schizotypy [30]. No previous study has examined the combined influence of specific polymorphisms related to brain dopamine activity and acute stress, as a gene-environment interaction, on impulsivity.

We designed a study to examine the complexity of the *ANKK1 *gene-environment interaction on impulsive behavior by testing acute stress as a plausible environmental factor in this relationship. To reduce the influence of potential confounds associated with psychopathology, we studied a community sample of young adults screened for psychiatric illness. Consistent with the multidimensional nature of impulsivity [31] the laboratory paradigm incorporated three separate measures of impulsivity, assessing reinforcer-cued approach, delay discounting and response disinhibition respectively. These three dimensions are supported by the results of factor analytic studies [32] and neuroimaging research linking these to differential brain activation patterns in areas connected to mesolimbic dopamine circuits. Reinforcement-related processing and delay discounting are linked to greater activation in the ventral striatum [33], while response inhibition is associated with orbitofrontal circuit activity [34]. We directly examined the relationship between the A1 allele, laboratory-induced acute psychosocial stress and laboratory measures of impulsive behavior.

## Methods

### Participants

The study was approved by the Queensland University of Technology Human Research Ethics Committee (Reference 3459H). All participants (44 females and 29 males) provided signed informed consent. Participants (*M *= 19.29 years, *SD *= 1.89) were recruited from technical college campuses through advertising. Potential participants were screened at initial contact via self-report for inclusion criteria: 17 to 25 years old, no history of head injury or psychiatric disorder, no current gum disease and sufficient English language to complete the questionnaires.

Of the 73 participants, 51 (69.9%) were Australian-born and 51 (69.9%) were of Caucasian/European ethnicity, with 6 (8.2%) reporting Polynesian ethnicity, 5 (6.8%) Asian ethnicity, 1 (1.4%) Aboriginal and/or Torres Strait Islander ethnicity and 9 (12.3%) reporting 'Other' ethnicity. On the highest level of education attained, 60 (82.2%) participants reported completing high school. Seven (9.6%) participants reported a forensic history, typically involving minor offences. Despite prior screening criteria, 2 (2.7%) participants reported a history of head injury in the demographic questionnaire, and 8 (11.0%) reported a history/prior diagnosis of a psychiatric disorder. However, all participants were assessed as having normal cognitive function by the Trail Making Test [35] and an absence of psychiatric symptoms according to the General Health Questionnaire-28 [36]. On this basis, their data were retained and used in subsequent analyses.

### Procedure

Participants were randomly allocated to either an experimental stress induction (preparation period for a video-taped speech) or relaxation induction condition (listening to relaxing music), with each induction lasting five minutes. This experimental manipulation consistently increases subjective feelings of stress and accompanying neuroendocrine and cardiovascular responses [37-39]. The experiment was conducted individually in order to maximize the effect of the psychosocial stressor and minimize social support confounds [40]. Specifically, those in the acute stress condition were told they were to spend the next five minutes preparing a speech on their least favorite body part which may be videotaped at the end of the testing session. These instructions are similar to those used successfully in previous research on the effect of alcohol on psychological stress [41,42] and the effect of psychosocial stress on decision-making performance [43]. A video camera was positioned on a tripod and visibly connected to the power supply in full view of the participant.

A behavioral measure of reinforcement sensitivity, the Card Arranging Reward Responsiveness Objective Test (CARROT) [44], was administered before and after the induction. This is a simple card-sorting task that measures over four trials the extent to which participants increase their speed of performance when reinforced via small financial rewards compared with non-reinforcement trials (see Table 1 for further details). The CARROT has sound validity as a behavioral measure of Gray's reinforcement sensitivity, with scores correlating with self-reported "reward sensitivity" in an Australian university sample [45]. Measures of state anxiety (State Trait Anxiety Inventory – State form, STAI-S) [46] and feelings of relaxation or stress via a 100 mm Visual Analogue Scale (VAS) measure were also administered pre and post. Participants were screened for psychopathology (General Health Questionnaire-28) [36] and adequate cognitive function (Trail Making Test) [35]. After questionnaire completion participants provided mouth swabs. Two computerized impulsivity tasks were administered after the induction and second CARROT administration. These were a forced-choice delay discounting task (choices paired with smaller, sooner-obtained point reinforcers vs. larger, longer delayed point reinforcers) named the Two Choice Impulsivity Paradigm (TCIP) [31] followed by a stop signal task assessing the ability to withhold a prepotent response, the GoStop task [31]. Due to the nature of the scoring of these tasks and the potential influence of carry-over practice effects, they could only be administered post-induction. Table 1 provides further detail on these behavioral measures of impulsivity.

**Table 1:** Procedure: Tests of behavioral impulsivity

Test	Description	Dependent Variable
CARROT [44]	Participants complete four trials of sorting a pack of cards, each card with five digits, into three corresponding trays. The first trial (T1) involves sorting 60 cards while being timed, with this time used as the time limit for subsequent trials. In trial two (T2), the participant sorts a pack of 100 cards until told to stop. The third trial (T3) involves sorting 100 cards again with the time restriction of the previous trial, but with a small monetary reward offered for every five cards correctly sorted. A 20 cent coin is placed in front of the participant as the fifth card is sorted into the correct trays. The fourth trial (T4) is identical to T2 and controls for fatigue or practice effects on response speed. After T4, the participant is given the money earned during T3.	CARROT score of reinforcement sensitivity, calculated by subtracting the mean of the number of cards sorted in T2 and T4 from the number of cards sorted in T3. CARROT = T3 - ((T2+T4)/2).
TCIP [31]	A forced-choice, reinforcement-directed computerized task, modeled on delay discounting and delay of gratification tasks. Participants press a mouse button to select one of two shapes (a square and a circle), each associated with either a short delay (in this case, 5 seconds) followed by a small reinforcer (in this case, 5 points) or a longer delay (15 seconds) followed by a larger reinforcer (15 points). For this experiment, the parameters were set to include 10 training trials followed by 40 session trials using the "Reward Feedback" option. Pairing of shapes with immediate/delayed conditions was counterbalanced within each experimental induction group. Reinforcement contingencies were not made explicit, with participants implicitly learning the relationship between the number of reward points displayed on the screen and each preceding geometric shape choice.	1. Proportion of more immediate reinforcer choices (higher = more impulsive) 2. Reaction times when making these more immediate reinforcer choices (faster = more impulsive).
GoStop [31]	Like other stop response inhibition procedures, participants are required to attend to a series of visual stimuli, respond when a target "go" signal appears, and withhold responding when a "stop" signal or non-target stimulus appears. In the GoStop, the stimuli are a series of five-digit numbers presented in black font one at a time on the screen. The "go" signal is a number that matches the previous number identically and is also presented in black. The "stop" signal is a matching number that changes color from black to red font some time after the stimulus onset. In addition to No-Stop (only the "go" signal) and Stop trials, at least half of the trials are Novel trials, with randomly generated non-matching numbers presented in black. For this experiment, the parameters were the default option of two blocks, seven stop trials (default is 10), 28 non-stop trials (default is 40), and 56 novel trials (minimum of one Novel stimulus following every Stop and No-Stop Trial). Stop Interval settings (ms from stimulus onset, SOA) were set as default (four intervals of 50 ms, 150 ms, 250 ms, and 350 ms, quasi-randomized throughout the session). Stimuli were presented for 500 ms each followed by 600 ms blackout between stimuli presentations.	1. Percent inhibited responses (proportion of Stop trials where no response occurs) (lower = more impulsive). 2. Stop Latency (time in ms between the Stop Signal onset and response) (quicker = more impulsive).

CARROT, Card Arranging Reward Responsiveness Objective Test; TCIP, Two Choice Impulsivity Paradigm.

### *Taq*IA Genotyping

Buccal mucosa cells were collected using *Cytosoft *brushes (Medical Packing Corporation, California, USA). Mouth swabs were used to obtain DNA samples to avoid a selective exclusion of participants with blood and injection phobias. These cells were spun and DNA was extracted from leucocytes using standard techniques and subsequently used as a template for determination of genotypes [47]. *ANKK1 Taq*IA genotyping was performed by restriction fragment length polymorphism (RFLP) analysis of PCR products. A genomic sequence of 501 bp of the coding region of *ANKK1 *was amplified by PCR using the forward primer 5'-GCACGTGCCACCATACCC-3' and the reverse primer 5'-TGCAGAGCAGTCAGGCTG-3'. A total of 5–10 ng of genomic DNA was amplified in a PCR master mix containing 0.2 μM of forward primer and 0.2 μM of reverse primer, 1× PCR buffer, 1.5 mM MgCl_2_, 200 μM dNTPs and 1 unit of Platinum *Taq *DNA Polymerase (Invitrogen) in a 25 μL volume. Amplification conditions were: Step 1: 94°C for 4 min, Step 2: 94°C for 30 s, Step 3: 68°C for 30 s, Step 4: 72°C for 30 s, Steps 2–4 were repeated by 40 cycles followed by 72°C for 3 min. Amplified PCR fragments were digested with *Taq*I restriction enzyme (New England Biolabs) and digested fragments were visualized via agarose gel electrophoresis.

*Taq*IA genotyping identified 45 (61.6%) participants as A2/A2 genotype (i.e., A1- allelic status), 24 (32.9%) as A1/A2 genotype and 4 (5.5%) as A1/A1 genotype (with the latter two genotypes classified as A1+ allelic status). These frequencies are in Hardy-Weinberg equilibrium, χ^2^(*N *= 73) = 0.11, *p *> 0.05. Subsequent analyses were performed comparing presence or absence of the A1 allele (A1+ vs. A1-). The gender and ethnicity distribution for these two genotypes are presented in Table 2. Chi-square analyses using Fisher's Exact Test showed no significant associations of allelic status (A1+ vs. A1-) with gender (*p *= 0.33) or with ethnicity (Caucasian vs. non-Caucasian, *p *= 0.999). Further, there were no significant main or interactive effects of gender or ethnicity (Caucasian vs. non-Caucasian) with genotype on the baseline impulsivity measure tested, *p *> 0.05.

**Table 2:** *ANKK1 Taq*IA A1 allele classification^a ^frequencies (% of total) by self-reported gender and ethnicity

Subgroup	A1+	A1-
Gender		
Male	9 (12.3%)	20 (27.4%)
Female	19 (26.0%)	25 (34.2%)
Total	28 (38.4%)	45 (61.6%)
Ethnicity		
Aboriginal and/or Torres Strait Islander	1 (1.4%)	0 (0%)
Caucasian/European	20 (27.8%)	31 (43.1%)
Polynesian	1 (1.4%)	5 (6.9%)
Asian	3 (4.2%)	2 (2.8%)
Other	3 (4.2%)	6 (8.3%)
Total	28 (38.9%)	44 (61.1%)

^a^A1+ participants have at least one A1 allele and A1- participants are homozygous for A2.

### Data Analyses

This experimental design was mixed (Time: pre, post × Genotype: A1A1 and A1A2 vs. A2A2 genotypes × Induction: stress, rest), with time as repeated measures for the CARROT only (and for manipulation checks involving the STAI and VAS measures). A square root transformation corrected a significant positive skew on TCIP mean choice latency for immediate reinforcers. Separate analyses of variance were conducted for each dependent variable of each task. While only pertinent results are reported, the full results of all analyses are available from the author.

## Results

### Experimental manipulation checks

Manipulation checks via paired t-tests on pre- and post-induction STAI-S scores confirmed the validity of the induction. Those exposed to the stress induction reported significantly more anxiety after exposure (*M *= 40.67, *SD *= 12.17) than at baseline (*M *= 36.78, *SD *= 10.75), *t*(35) = -2.50, *p *= 0.017. Those exposed to the relaxation induction significantly reduced their anxiety scores from baseline (*M *= 37.35, *SD *= 11.72) to post-induction (*M *= 31.24, *SD *= 8.15), *t*(36) = 4.59, *p *< 0.001. Further tests revealed no baseline differences between the two induction groups, *t*(71) = 0.22, *p *= 0.83, and no effect of *Taq*IA genotype on anxiety scores at baseline or over time (*p *> 0.05). These test results were replicated using the VAS measure of stress, further supporting the validity of the stress manipulation.

### Tobacco smoking status confound check

A self-report measure of tobacco smoking showed 27 (37.0%) of the sample were current smokers. A chi-square analysis of smoking status by *Taq*IA allelic status by induction condition using Fisher's exact Test showed no significant association of smoking status with allelic status for either the rest induction (*p *= 0.9999) or stress induction (*p *= 0.50) groups.

### Correlations between impulsivity measures

Table 3 presents the intercorrelations between the three laboratory paradigms used to measure impulsivity, namely the CARROT, TCIP and GoStop task variables. As shown, correlations between the three paradigms are all low, supporting their relative independence.

**Table 3:** Intercorrelations between laboratory measures of impulsivity

	1	2	3	4	5	6	7	8	9	10	11	12
1. CARROT1	-	-.02	.10	.08	.04	-.13	-.01	.03	-.02	.13	.10	.02
2. CARROT2		-	.01	.00	.04	-.05	-.08	.05	-.14	.17	.05	-.01

3. TCIP PrChIm			-	-.68**	.07	-.01	.09	.04	.07	.00	.05	-.05
4. TCIP MChLIm				-	-.07	.09	.04	.05	.11	-.16	-.06	.08

5. GS St50Inpc					-	.71**	.39**	.28*	.39**	.51**	.46**	.74**
6. GS St150Inpc						-	.54**	.37**	.36**	.34**	.32**	.67**
7. GS St250Inpc							-	.58**	.31*	.20	.14	.52**
8. GS St350Inpc								-	.19	.07	.11	.28*
9. GS St50StL									-	.42**	.44**	.60**
10. GS St150StL										-	.62**	.68**
11. GS St250StL											-	.68**
12. GS St350StL												-

*Note*. CARROT = Card Arranging Reward Responsiveness Objective Test score – first (1) and second administration (2); TCIP = Two Choice Impulsivity Paradigm, PrChIm = Proportion of Immediate Choices made, MChLIm = Mean choice latency (ms) to choose the option paired with an 'immediate' reinforcer, from presentation of stimuli to response; GS = GoStop task, St[50]Inpc = % inhibition at [50], [150], [250], and [350] ms SOAs; St[50]StL = response latency on Stop trials at [50], [150], [250], and [350] ms SOAs.

** Correlation is significant at the 0.01 level (2-tailed). * Correlation is significant at the 0.05 level (2-tailed).

### Reinforcement sensitivity: CARROT scores

**Figure 1:**
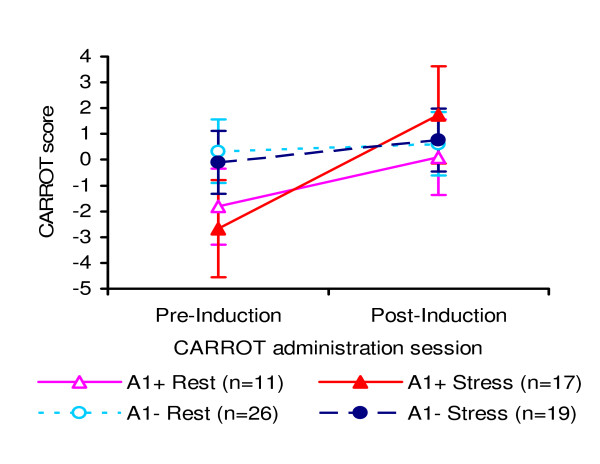
**Reward sensitivity (CARROT scores) pre- and post-induction (rest vs. stress) by *ANKK1 Taq*IA allelic status (A1+ vs. A1-)**. Error bars display ± 2 *SEM*.

A Genotype × Induction × Time split-plot ANOVA revealed a multivariate two-way interaction between time and genotype on CARROT scores, *F*(1,69) = 5.41, *p *= 0.023, *η*_*p*_^2 ^= 0.073. Further comparisons revealed that at baseline, A1+ participants were slower in card sorting under reinforcement conditions versus non-reinforcement conditions, compared with A1- participants, *F*(1,71) = 6.98, *p *= 0.010, *η*_*p*_^2 ^= 0.089. These CARROT scores improved for A1+ participants after exposure to either induction (*F*(1,71) = 16.02, *p *< 0.001, *η*_*p*_^2 ^= 0.184), but not for A1- participants (*F*(1,71) = .62, *p *= 0.43, *η*_*p*_^2 ^= 0.009) (Figure 1). There were no interactive or main effects of the stress manipulation (*p *> 0.05).

### Delay discounting: TCIP measures

Separate Genotype × Induction between-groups ANOVAs were conducted for TCIP measures, proportion of smaller sooner 'immediate' reinforcer choices made and square root transformed mean response latencies for 'immediate' reinforcer selections (Table 4). There were no significant effects of allelic status or the stress manipulation (*p *> 0.05).

**Table 4:** TCIP Delay Discounting by *ANKK1 Taq*IA genotype and induction condition (Rest A1+ *n *= 11, A1- *n *= 25; Acute Stress A1+ *n *= 17, A1- *n *= 18)

	Proportion Impulsive Choices		Mean Immediate Choice Latency^a ^(ms)	
	
Induction Condition and Allele	*M*	*SD*	*M*	*SD*
Rest				
A1+	0.34	0.22	2500.73	1094.28
A1-	0.39	0.28	2515.80	1283.48
Stress				
A1+	0.36	0.20	2220.12	900.13
A1-	0.46	0.30	2223.33	804.80

^a^Means based on untransformed raw latencies.

### Inhibitory control: GoStop measures

**Figure 2:**
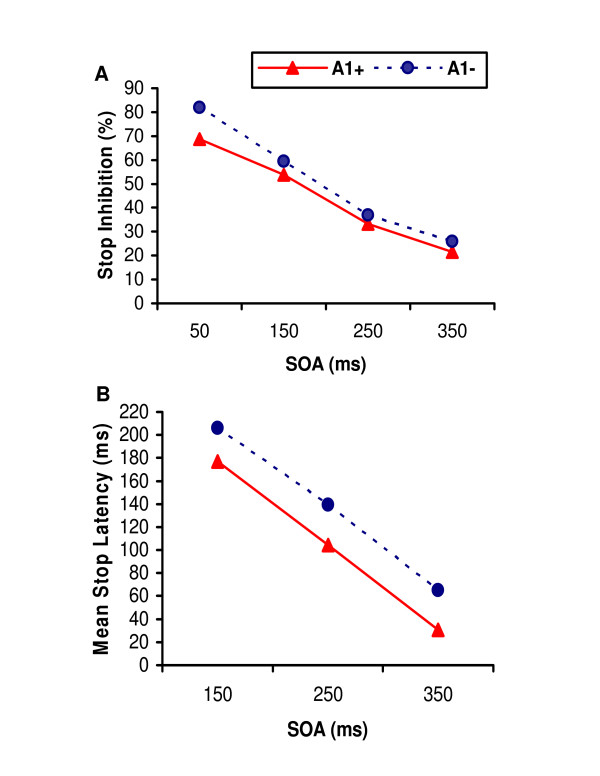
(A) Stop inhibition (%) and (B) mean response latency on stop trials (ms) of *ANKK1 Taq*IA allelic groups (A1+ vs. A1-), as a function of stimulus onset asynchrony (SOA, ms) of the stop signal.

We examined the interaction and main effects of the A1 allele and stress exposure via separate mixed-design ANOVAs for the two indices of rash impulsiveness, poorer stop inhibition and faster mean response latencies when responding to the stop signal. Mean latency data at 50 ms SOA (stimulus onset asynchrony) contained 21% missing data (15 participants successfully inhibited the prepotent response 100% of the time) and was thus excluded from analysis. For both measures, main effects of *Taq*IA genotype emerged, with A1+ participants demonstrating poorer stop inhibition (*F*(1,68) = 4.22, *p *= 0.04, *η*_*p*_^2 ^= 0.058) and quicker mean latencies on stop trials (*F*(1,66) = 9.45, *p *= 0.003, *η*_*p*_^2 ^= 0.125) than A1- participants across all SOAs (Figure 2). Again, there were no interactive or main effects of the stress manipulation (*p *> 0.05). There were consistent effects of SOA on both response inhibition (*F*(4,66) = 90.52, *p *< 0.001, *η*_*p*_^2 ^= 0.80) and mean stop response latency (*F*(2,65) = 288.69, *p *< 0.0001, *η*_*p*_^2 ^= 0.90), with poorer stop inhibition and faster incorrect responses as SOA increased.

## Discussion

Our study has, for the first time, examined several behavioral measures of impulsivity in combination with molecular genetic risk and acute psychosocial stress. We have shown that the *Taq*IA A1 allele is associated with preclinical behavioral risk in two dimensions of impulsivity, one characterized by impaired performance sensitive to reinforcers in the environment (though quickly overcome with repeated exposure) and the other by an accelerated and disinhibited approach style in the absence of incentive cues. This shared genetic risk was not associated with acute stress and may represent indirect evidence of a common etiology involving altered brain dopaminergic activity, consistent with reward deficiency theory [2,5].

Similar reinforcement-related performance deficits associated with A1+ allelic status have been previously reversed under the influence of the dopamine agonist, bromocriptine [48]. While an acute stressor can increase dopamine neurotransmission in humans [29], our study has shown a baseline performance deficit and subsequent improvement for A1+ participants regardless of acute stress. These results may reflect the importance of dopaminergic activity in the reinforcement pathways for initial CARROT performance and an increased sensitivity of those with deficiencies conferred by the A1 allele to repeated cue exposure. The second finding of poorer response inhibition by A1+ participants has been shown in Spanish alcohol dependent male patients [49]. Our extension of these findings to a non-clinical sample comprising both male and female young adults provides strong support for an association of the A1 allele with an impulsive endophenotype, rather than as a confound with alcoholism. The combined rash impulsive profile shown on this task by A1+ young adults is also similar to that shown previously for adults diagnosed with ADHD, a disorder characterized by impulsivity [50].

We suggest the A1 allele confers a greater need for practice to overcome inherent reinforcement-related learning deficits associated with fewer dopamine receptors in key brain reinforcement sites. Once sensitized, the general acceleration of approach behavior may be associated with reduced inhibitory control observed in A1+ participants. Slower baseline approach in the presence of financial reinforcers could alternatively reflect an attentional bias towards reinforcement cues in the environment, thus indicating heightened reinforcement sensitivity in these individuals. This alternative is consistent with reward deficiency [2,5].

The absence of *Taq*IA effects on implicit delay discounting contrasts with the impulsive profile suggested by effects on the other two behavioral measures. This may reflect a distinction between implicit and explicit awareness of reinforcers, given a contrasting recent report of an association between the A1 allele and an explicit delay discounting paradigm in an unselected university sample [51]. Future research should further examine a comparison between explicit and implicit cognitive processes with the A1 allele.

The absence of an effect of the acute psychosocial stressor on the *ANKK1 Taq*IA-impulsivity relationship, despite a significant increase in subjective anxiety, underscores the importance of the *ANKK1 *main effect on impulsivity. Further, while our results need to be replicated with a larger sample, they show that acute psychosocial stress is an unlikely explanation of why some individuals with the A1 allele exhibit impulsive tendencies. The addition of physiological and neuroendocrine stress measurement in future research is important. Future research should also investigate other plausible environmental moderators and mediators of this relationship, including chronic stress.

## Conclusion

Our findings have helped elucidate the nature of an impulsivity endophenotype associated with the A1 allele of the *ANKK1 Taq*IA polymorphism that is differentially related to a rash impulsive behavioral style and reinforcement-related performance deficits. This phenotype is unaffected by acute stress exposure.

## Competing interests

The authors declare that they have no competing interests.

## Authors' contributions

MW participated in the design of the study and coordination, carried out the experiments, performed the statistical analyses and drafted the manuscript as part of a PhD thesis. CM participated in the design and coordinated the DNA extraction and genotyping. BL participated in the design. BL and CM were PhD co-supervisors. RY participated in the design and coordination, helped to draft the manuscript and was primary PhD supervisor. All authors read and approved the final manuscript.
